# CSF proteomic profiles related to cognitive decline in MCI A+ depend on tau levels

**DOI:** 10.1093/brain/awaf251

**Published:** 2025-07-08

**Authors:** Eleonora M Vromen, Diederick M de Leeuw, Argonde C van Harten, Charlotte E Teunissen, Wiesje M van der Flier, Pieter Jelle Visser, Betty M Tijms

**Affiliations:** Alzheimer Center Amsterdam, Neurology, Vrije Universiteit Amsterdam, Amsterdam UMC Location VUmc, Amsterdam, North-Holland 1081HZ, The Netherlands; Amsterdam Neuroscience, Neurodegeneration, Amsterdam, North-Holland 1081HZ, The Netherlands; Alzheimer Center Amsterdam, Neurology, Vrije Universiteit Amsterdam, Amsterdam UMC Location VUmc, Amsterdam, North-Holland 1081HZ, The Netherlands; Amsterdam Neuroscience, Neurodegeneration, Amsterdam, North-Holland 1081HZ, The Netherlands; Alzheimer Center Amsterdam, Neurology, Vrije Universiteit Amsterdam, Amsterdam UMC Location VUmc, Amsterdam, North-Holland 1081HZ, The Netherlands; Amsterdam Neuroscience, Neurodegeneration, Amsterdam, North-Holland 1081HZ, The Netherlands; Amsterdam Neuroscience, Neurodegeneration, Amsterdam, North-Holland 1081HZ, The Netherlands; Neurochemistry Laboratory, Department of Clinical Chemistry, Vrije Universiteit Amsterdam, Amsterdam UMC Location VUmc, Amsterdam, North-Holland 1081HZ, The Netherlands; Alzheimer Center Amsterdam, Neurology, Vrije Universiteit Amsterdam, Amsterdam UMC Location VUmc, Amsterdam, North-Holland 1081HZ, The Netherlands; Amsterdam Neuroscience, Neurodegeneration, Amsterdam, North-Holland 1081HZ, The Netherlands; Alzheimer Center Amsterdam, Neurology, Vrije Universiteit Amsterdam, Amsterdam UMC Location VUmc, Amsterdam, North-Holland 1081HZ, The Netherlands; Amsterdam Neuroscience, Neurodegeneration, Amsterdam, North-Holland 1081HZ, The Netherlands; Alzheimer Centrum Limburg, Department of Psychiatry, Maastricht University, Maastricht, Limburg 6200 MD, The Netherlands; Department of Neurobiology, Care Sciences and Society, Division of Neurogeriatrics, Karolinska Institutet, Stockholm 171 77, Sweden; Alzheimer Center Amsterdam, Neurology, Vrije Universiteit Amsterdam, Amsterdam UMC Location VUmc, Amsterdam, North-Holland 1081HZ, The Netherlands; Amsterdam Neuroscience, Neurodegeneration, Amsterdam, North-Holland 1081HZ, The Netherlands

**Keywords:** CSF proteomics, Alzheimer disease, mild cognitive impairment, cognitive decline

## Abstract

Individuals with mild cognitive impairment (MCI) and an abnormal amyloid biomarker (A+) are at considerable increased risk of developing dementia. Still, these individuals vary greatly in rates of cognitive decline, and the mechanisms underlying this heterogeneity remain largely unclear. One factor related to an increased risk of progression to dementia is having an abnormal tau status (T+), but this still explains only part of the variance. Furthermore, previous work has indicated that MCI A+ individuals with T– or T+ are characterized by distinct molecular processes as reflected by distinct CSF proteomic profiles. As such, it could be hypothesized that differences in rates of cognitive decline in A+ MCI with abnormal or normal tau status may be explained by distinct underlying mechanisms. We studied this question using an untargeted CSF proteomic approach in individuals with MCI and abnormal amyloid.

We measured untargeted Tandem Mass Tag (TMT) mass spectrometry proteomics in CSF of 80 A+ MCI individuals from the Amsterdam Dementia Cohort [age 66 ± 7.9 years, 52 (65%) T+]. For each protein, we tested if CSF levels were related to time to progression to dementia using Cox survival models; and with decline on the Mini-Mental State Examination (MMSE) with linear mixed models, correcting for age, sex and education. We validated our results in the independent Alzheimer’s Disease Neuroimaging Initiative (ADNI) that employed the orthogonal CSF Soma logic protein measures in 245 CSF A+ MCI individuals [age 73 ± 7.2 years, 135 (55%) T+].

In total, we found 664 (29%) proteins to be related to cognitive decline in A+T+ and 718 (31%) proteins in A+T–. In A+T+, higher levels of 393 proteins that were associated with synaptic plasticity processes, and lower levels of 271 proteins associated with the immune function processes predicted a steeper decline on the MMSE and faster progression to dementia. In A+T−, higher levels of 306 proteins that were related to blood–brain barrier impairment and lower levels of 412 proteins associated with synaptic plasticity processes predicted a steeper decline; 67% of pathways associated with a decline in A+T+ and 58% in A+T– were replicated in ADNI.

In conclusion, cognitive decline in A+ MCI individuals with and without tau may involve distinct underlying pathophysiology. These findings suggest that treatments aiming to delay cognitive decline may need tailoring according to the underlying mechanism of these patient groups, and that amyloid and tau levels could aid in stratification of selecting patients.

## Teaser

Vromen *et al.* show that rates of cognitive decline in people with mild cognitive impairment and amyloid positivity (MCI A+) are associated with distinct proteomic signatures depending on whether or not abnormal tau status is also present.

## Introduction

Alzheimer’s disease (AD) is a neurodegenerative disorder and the most common cause of dementia.^1^  Ad is characterized by pathological depositions of amyloid-β (A) and hyperphosphorylated tau (T) in the brain, which can be detected *in vivo* by abnormal CSF levels of these proteins.^2,3^ Individuals with mild cognitive impairment (MCI) and abnormal amyloid (A+) are at increased risk of developing dementia,^4,5^ but individuals vary in their rates of cognitive decline. One factor related to a steeper decline in MCI A+ is the presence of an abnormal tau biomarker.^4-6^ Still, even within MCI A+T+ rates of disease progression remain highly variable,^7^ and other factors like sex, age or level of education explain only part of the variability. More knowledge on underlying mechanisms is relevant to work towards clinically effective therapeutic drugs that slow or stop cognitive decline.

One possibility is that, in addition to amyloid and tau, other biological processes may be involved in disease progression. One approach to studying such biological processes is through proteomic measures in CSF. Taking such an approach, we previously found a proteomic signature with the Olink proximity extension assay that predicted cognitive decline in MCI A+ and which was associated with immune system processes, signal transduction and neuronal death.^8^ However, in that study, it remained unclear to what extent the processes related to cognitive decline in MCI A+ may depend on tau status, as the sample size was too small for such stratified analyses. Possibly, the difference in rates of decline within MCI individuals with A+T– and A+T+ might be in part related to different underlying biological processes. We and others previously observed that individuals with A+T– or A+T+ were characterized by distinct CSF proteomic signatures.^9,10^ Individuals with A+T+ biomarker profiles were characterized by mostly increased levels of proteins that were related to neuronal plasticity. A similar proteomic signature has also been observed in tissue studies with worse tau pathology as indicated by pathological Braak scores.^9,11-15^ Furthermore, these same proteins that had increased CSF levels in A+T+ individuals were decreased in individuals with A+T−, suggesting that those individuals may have neuronal hypo-plasticity. In addition, A+T– individuals were characterized by increased levels of proteins that were indicative of blood–brain barrier dysfunction.^9,15,16^ Together, these results led us to hypothesize that different types of biological processes may underlie cognitive decline in MCI A+T– and MCI A+T+. To study this question, large sample sizes of MCI individuals with CSF and clinical follow-up were required, which we were able to obtain from the Amsterdam Dementia Cohort (ADC). Furthermore, an untargeted mass spectrometry approach could capture additional proteins that may be linked to decline, which were not included in earlier research that employed a targeted approach, to explore proteomics in CSF in relation to cognitive decline.^9^

In this study, we investigated whether variability in cognitive decline in MCI A+ is associated with distinct biological processes as reflected in CSF proteomics and to what extent such associations differed depending on tau status. We repeated the analyses in the ADNI study as an independent replication cohort, employing an orthogonal proteomic approach. We modelled cognitive decline by studying time to progression to dementia as well as changes over time on the Mini-Mental State Examination (MMSE) as a continuous measure for progression that may have more power to capture decline for those individuals at risk who have not yet reached a clinical dementia status within the follow-up period.

## Materials and methods

### Participants

In this study we analysed two cohorts: the ADC for the main analyses and the Alzheimer’s Disease Neuroimaging Initiative (ADNI) cohort for replication analyses. From the ADC, 80 individuals with MCI and an abnormal CSF amyloid marker were selected based on available CSF proteomics and clinical follow-up. Repeated MMSE measurements were available for 77 (96%) MCI individuals [mean ± standard deviation (SD) 4.6 ± 1.9 measurements over 4.49 ± 2.1 years]. The ADC is a clinical cohort that consists of individuals who visited the memory clinic of the Alzheimer Center Amsterdam.^17^ Most individuals visiting the memory clinic received extensive routine diagnostic work-up, usually including, amongst others, neurological examination, neuropsychological testing and CSF sampling. A diagnosis of MCI was based on international consensus guidelines^18,19^ and made by consensus during a multidisciplinary meeting. Individuals received clinical follow-up, including neuropsychological testing, at approximately 12 months, at which time the clinical diagnosis could be changed to dementia if international consensus criteria were met.^20,21^ For validation purposes, we selected 245 CSF A+ MCI individuals from the ADNI, based on the same inclusion criteria as the main study. In ADNI, the mean ± SD follow-up duration was 4.8 ± 3.2 years. All individuals had repeated MMSE measurements available (6.2 ± 2.6 tests). The ADNI (adni.loni.usc.edu) is a public–private partnership that was launched in 2003 and is led by Principal Investigator Michael W. Weiner. ADNI’s main aim has been to investigate MCI and early AD dementia and to investigate whether progression can be measured using MRI, PET, CSF biomarkers and neurological and neuropsychological assessments that are routinely collected. Up-to-date information can be found on adni-info.org. For ADNI, diagnoses of MCI and dementia were based on clinical guidelines as previously described.^18,20,22-24^ We also included 103 controls with normal cognition and normal CSF amyloid and tau markers from ADC and 84 from ADNI to serve as a reference group to normalize protein levels, so that interpretation of the results was comparable across cohorts (see next section for details).

### CSF collection and measurements

A lumbar puncture was performed to collect CSF following routine procedures.^25^ CSF was processed and stored as previously described.^26,27^ In the ADC, Aβ 1–42 (Aβ_42_), phosphorylated tau_181_ (p-tau) and total tau (t-tau) concentrations were determined with ELISAs from Innotest (β-amyloid1-42, hTAU-Ag and PhosphoTAU-181p, Fujirebio). The previously published cut-offs of drift-corrected Aβ_42_ < 813 pg/ml and t-tau >375 pg/ml were used to determine, respectively, amyloid and tau abnormality.^28,29^ In ADNI, Aβ_42_, p-tau and t-tau concentrations were determined with the Luminex xMAP platform (Luminex Corp) with the INNO-BIA AlzBio3 kit (Fujirebio).^30^ The cut-offs were <192 pg/ml and >93 pg/ml, respectively.^31^ T-tau was preferred over p-tau as this was available in more participants and correlated highly (correlation of p-tau and t-tau in ADC: *r* = 0.95, *P*-value <0.001; in ADNI: *r* = 0.65, *P*-value <0.001).

In the ADC, CSF proteomics were measured at baseline with liquid chromatography-tandem mass spectrometry (LC-MS/MS) in an untargeted manner based on tandem mass tag (TMT) labelling with 16-plexing, using a high pH reverse phase high-performance liquid chromatography (HPLC) for peptide prefractionation, as described in detail previously.^15^ We used reference channels to normalize relative peptide abundances between TMT plex experiments, according to standard procedures.^32^ In total, 3863 unique proteins were detected. Proteins were included in our analysis when they were detected in at least 50% of individuals, resulting in 2302 proteins, of which the vast majority were measured in 100% of individuals. In ADNI, targeted CSF proteomics were measured on the SOMAscan platform (SOMAscan7k). In total, 7008 aptamers were measured, corresponding to 6164 unique protein targets, and all were available in at least 50% of individuals. Data normalization and quality control were performed by SOMAlogic. Details are described elsewhere.^33^ In both cohorts, protein measures were log-transformed and then scaled (*Z*-transformed) to the mean and standard deviation of the control group. In total, 1511 proteins were detected in both cohorts.

### Statistical analysis

All statistical analyses were performed in R version 4.2.1—‘Funny-Looking Kid’.^34^ Demographics were described and compared between A+T+ and A+T– using standard statistical measures. We first tested differences between AT groups in decline on the MMSE over time using linear mixed models (LMMs), as well as differences between AT groups in clinical progression to dementia with Cox proportional hazard models. Next, for each T group separately, we tested which proteins in the CSF levels at baseline were associated with subsequent cognitive decline over time in the following ways: (i) using LMMs, we tested associations between baseline protein levels and time as main terms and together as an interaction term to test changes over time on the MMSE; and (ii) using Cox proportional models, we tested the associations between baseline protein levels and time to progression to dementia. All models were adjusted for age, sex and level of education. LMMs also included random intercepts for patients, and fixed slopes. When at least 10 proteins were associated with cognitive decline on one or more outcome measures [*P*-values < 0.05, because we hypothesized that specific biological processes may be related to cognitive decline and in order to detect these pathways no false discovery rate (FDR) correction was applied at the protein level], pathway enrichment analysis was performed using Gene Ontology (GO)^35,36^ Biological Processes as part of the PANTHER classification system^37^ and adjusted for multiple testing with the false discovery procedure (pFDR < 0.05). We further annotated proteins as indicative of blood–brain barrier dysfunction according to Dayon *et al*.^38^ We repeated our analysis in the ADNI cohort to investigate whether we could replicate pathways associated with cognitive decline using the GO term at ID level.

### Ethical approval

This study was approved by the Ethics Committee of the Amsterdam UMC location VUmc and the Biobank Research Ethics Committee of the Amsterdam UMC location VUmc. All participants or surrogates provided written informed consent.

## Results

### Demographics and cognition

**Figure 1:**
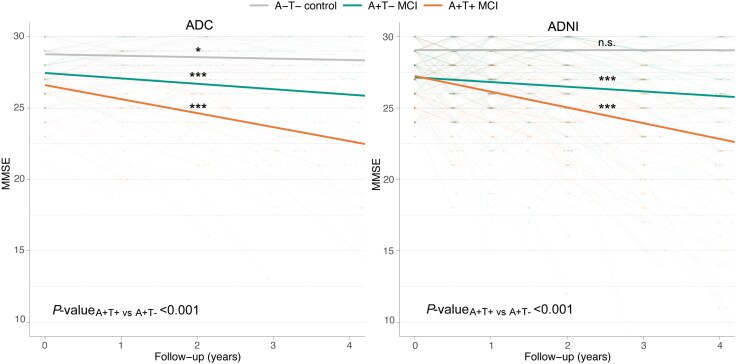
**MMSE trajectory over time in A+T+ and A+T– MCI individuals from the ADC and ADNI cohorts.** MMSE scores in A+T+ MCI (orange), A+T− MCI (green) and A–T– cognitively normal (grey) individuals from the Amsterdam Dementia Cohort (ADC; *left*) and Alzheimer's Disease Neuroimaging Initiative (ADNI; *right*). The *P*-value for each group slope (compared with 0) is indicated just above or below each line. ***P-value <0.001, **P-value <0.01, *P-value <0.05. AT = CSF amyloid and tau status; MCI = mild cognitive impairment; MMSE = Mini-Mental State Examination; n.s. = not significant.

In total, 80 A+ MCI individuals were included from the ADC [age 66.4 ± 7.9 years, 28 (35%) female, 52 (65%) A+T+; Table 1]. The ADNI validation cohort included 245 A+ MCI individuals [age 73.2 ± 7.2 years, 102 (42%) female, 135 (55%) A+T+; Table 1]. MMSE scores at baseline were lower in A+T+ than A+T– in both cohorts (ADC: A+T+ versus A+T−: 26.3 ± 2.0 versus 27.2 ± 1.6, *P*-value = 0.044; ADNI: A+T+ versus A+T−: 27.2 ± 1.8 versus 27.7 ± 1.8, *P*-value = 0.039) and for both AT groups lower than controls (both cohorts *P*-value < 0.001). Both AT groups declined faster over time on the MMSE compared with controls, with a steeper decline in A+T+ (ADC: β ± SE −0.99 ± 0.08; ADNI: β ± SE −1.11 ± 0.04, *P*-values <0.001) than in A+T– individuals (ADC: β ± SE −0.39 ± 0.09; ADNI: β ± SE −0.35 ± 0.04, *P*-values < 0.001, *P*-values_A+T+_  _versus A+T−_ < 0.001; Fig. 1). Individuals with A+T+ had an increased risk of progression to dementia compared with A+T– individuals, albeit not significant in the ADC [ADC: hazard ratio (HR) 1.7, 95% confidence interval (CI): 0.83–3.49, *P*-value = 0.149; ADNI: HR 1.85, 95% CI: 1.28–2.68, *P*-value = 0.001].

**Table 1:** Cohort demographics

–	Main cohort (ADC)						Replication cohort (ADNI)					
	Control	MCI—all	*P*-value Control versus MCI	MCI A+T–	MCI A+T+	*P*-value A+T– versus A+T+	Control	MCI—all	*P*-value Control versus MCI	MCI A+T–	MCI A+T+	*P*-value A+T– versus A+T+
*n*	103	80	–	28	52	–	84	245	–	110	135	–
Age at baseline (years), mean ± SD	58.80 ± 7.75	66.35 ± 7.89	**<0**.**001**	64.57 ± 8.69	67.31 ± 7.33	0.140	73.28 ± 5.38	73.15 ± 7.20	0.877	73.49 ± 6.82	72.87 ± 7.52	0.51
Women, *n* (%)	33 (32.0)	28 (35.0)	0.792	7 (25.0)	21 (40.4)	0.258	37 (44.0)	102 (41.6)	0.796	32 (29.1)	70 (51.9)	**0**.**001**
Education (years), mean ± SD	12.26 ± 2.94	12.35 ± 3.19	0.849	13.04 ± 3.17	11.98 ± 3.18	0.161	16.32 ± 2.76	15.91 ± 2.94	0.258	16.06 ± 3.03	15.78 ± 2.88	0.451
APOE e4-carrier, *n* (%)	32 (31.7)	55 (71.4)	**<0**.**001**	16 (59.3)	39 (78.0)	0.141	10 (11.9)	167 (68.2)	**<0**.**001**	65 (59.1)	102 (75.6)	**0**.**009**
CSF p-tau (pg/ml), mean ± SD	39.39 ± 12.62	75.78 ± 34.34	**<0**.**001**	44.61 ± 15.06	92.56 ± 29.76	<0.001	24.34 ± 11.78	48.32 ± 24.35	**<0**.**001**	34.89 ± 17.28	59.05 ± 23.90	**<0**.**001**
CSF t-tau (pg/ml), mean ± SD	220.31 ± 69.41	554.85 ± 335.76	**<0**.**001**	263.82 ± 88.28	711.56 ± 314.58	<0.001	55.01 ± 16.45	112.94 ± 58.56	**<0**.**001**	65.07 ± 18.48	151.95 ± 50.52	**<0**.**001**
Length of follow-up (years), mean ± SD	3.97 ± 3.22	3.97 ± 1.87	0.994	4.36 ± 2.14	3.75 ± 1.67	0.166	6.75 ± 4	4.17 ± 2.8	**<0**.**001**	4.5 ± 3.1	3.9 ± 2.5	**0**.**101**
Progression to AD-dementia, *n* (%)	–	–	–	11 (39.3)	33 (63.5)	0.066	10 (11.9)	133 (54.3)	**<0**.**001**	46 (41.8)	87 (64.4)	**0**.**001**
Progressors: time to progression (years), mean ± SD	–	–	–	2.47 ± 1.07	2.63 ± 1.51	0.692	6.61 ± 4.5	2.69 ± 2.3	**0**.**023**	2.55 ± 2.5	2.76 ± 2.3	0.642
Non-progressors: total follow-up time (years), mean ± SD	–	–	–	4.02 ± 2.68	3.04 ± 1.39	0.204	6.19 ± 3.6	3.76 ± 2.8	**<0**.**001**	4.39 ± 3.3	2.92 ± 1.8	**0**.**003**
*n* with longitudinal MMSE	103	77	–	28	49	–	84	245	–	110	135	–
MMSE at baseline, mean ± SD	28.41 ± 1.48	26.64 ± 1.91	**<0**.**001**	27.21 ± 1.64	26.31 ± 1.99	**0**.**044**	29.12 ± 1.09	27.44 ± 1.84	**<0**.**001**	27.71 ± 1.83	27.22 ± 1.81	**0**.**039**
Number of repeated MMSE per individual, mean ± SD	3.71 ± 1.79	4.52 ± 2.03	**0**.**005**	4.50 ± 1.93	4.53 ± 2.10	0.950	6.82 ± 2.9	6.04 ± 2.5	**0**.**027**	6.22 ± 2.6	5.9 ± 2.3	0.316

Controls were defined as cognitive normal individuals with normal amyloid and tau (AT) biomarkers. ADC = Amsterdam Dementia Cohort; ADNI = Alzheimer’s Disease Neuroimaging Initiative; MCI = mild cognitive impairment; AD = Alzheimer’s disease; MMSE = Mini-Mental State Examination.

### CSF proteome associations with MMSE score—at baseline and over time

**Figure 2:**
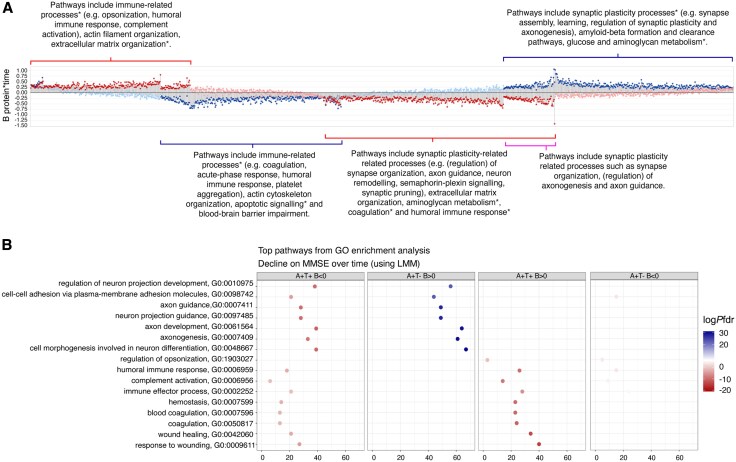
**Illustration of which proteins and their associated biological processes are related to cognitive decline in A+T+ and A+T−.** (A) Lollipop plot showing each protein associated with decline on MMSE over time in A+T+ (red) and A+T− (blue) MCI individuals. Each dot corresponds to a β estimate for the Protein × Time effect. Darker colour indicates that the Protein × Time was significant (*P*-value < 0.05), while lighter colour indicates that the Protein × Time was not significant. β-values > 0 indicate that lower protein levels were associated with a steeper decline, while β-values > 0 indicate that higher protein levels were associated with a steeper decline over time. Analyses were age, sex and education adjusted. An asterisk (*) behind a pathway indicates that the pathway was replicated in the Alzheimer's Disease Neuroimaging Initiative (ADNI) cohort. AT = CSF amyloid and tau status; β = β estimate; MCI = mild cognitive impairment; MMSE = Mini-Mental State Examination. (**B**) Results from Gene Ontology (GO) pathway enrichment analysis on proteins associated with decline on MMSE over time in A+T+ and A+T−, stratified for direction of effect. GO:BP was used as the ontology source. Pathways with the highest *P*-value false discovery rate (pFDR) are shown. AT = CSF amyloid and tau status; GO:BP = Gene Ontology Biological Processes; HR = hazard ratio.

We first investigated which protein CSF levels at baseline were associated with changes over time on the MMSE in A+T+ and A+T– individuals. Detailed statistics are described for each of the proteins in Supplementary Table 1 for all results, here we summarize the main findings. We observed a large number of proteins that were associated with longitudinal changes in the MMSE that were largely distinct for each biomarker group [A+T+: 664 (29%) proteins, A+T−: 718 (31%) proteins; Fig. 2A and Supplementary Table 1]. In A+T+, higher levels of 393 of 664 proteins were associated with a steeper decline on MMSE scores. Top pathways from the enrichment analysis included processes related to synaptic plasticity (e.g. synapse organization, axon guidance, neuron remodelling, semaphorin-plexin signalling and synaptic pruning) as well as pathways related to extra cellular matrix (ECM) organization, aminoglycan metabolism, coagulation and humoral immune response (Fig. 2B and Supplementary Table 2). Furthermore, for the other 271 of 664 proteins we observed that lower CSF levels were associated with a steeper decline, and were enriched for pathways involved in the immune system (e.g. opsonization, humoral- and immunoglobulin-mediated immune response and the lectin and alternative pathway of complement activation), but also actin filament organization and ECM organization.

**Figure 3:**
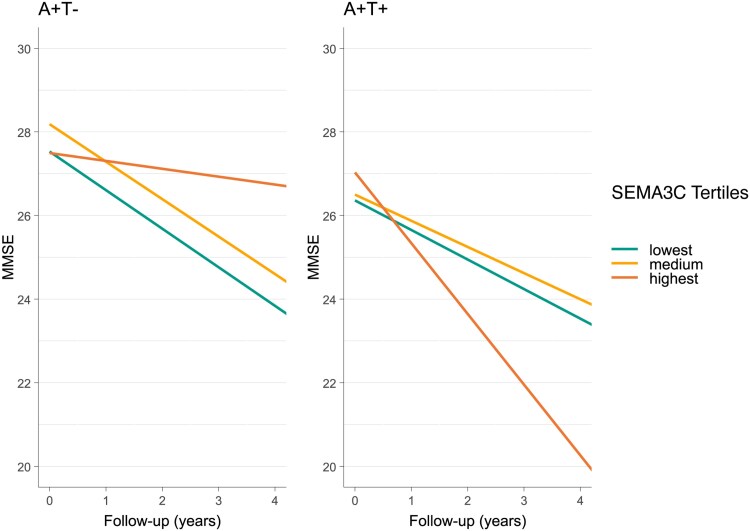
**Plot illustrating how proteins may have an opposite effect on MMSE score over time (i.e. Protein × Time β > 0 in A+T+ and < 0 in in A+T– or vice versa).** For this plot, SEMA3C levels were divided into tertiles across the total group, and MMSE scores over time were predicted for each tertile stratified for T status. *Left*: A+T+ MCI individuals and *right*: A+T– MCI individuals. AT = CSF amyloid and tau status; MCI = mild cognitive impairment; MMSE = Mini-Mental State Examination; SEMA3C = Semaphorin 3C.

In A+T−, higher levels of 306 of 718 proteins were associated with a steeper decline on the MMSE. These proteins were involved in immune-related processes including coagulation, acute-phase response and platelet aggregation, that was also associated with a decline in the A+T+ group. However, in the A+T– group these pathways were mostly driven by other proteins than in the T+ group, suggesting that distinct aspects of these processes are related to cognitive decline depending on T status. We further observed that the proteins related to decline in this group were enriched for actin cytoskeleton organization, apoptotic signalling and showed an overrepresentation of proteins that was suggestive of blood–brain barrier impairment (*n* = 17 out of 74 proteins indicative of blood–brain barrier dysfunction, *P*-value = 0.014). For the other 412 of 718 proteins we found that lower levels were related to a steeper decline of the MMSE in A+T−. These proteins were associated with synaptic plasticity-related processes (including synapse assembly, learning, regulation of synaptic plasticity and axonogenesis), as well as processes related to amyloid β formation and clearance, glucose metabolism and aminoglycan metabolism. A total of 87 of these 412 proteins for which lower levels in A+T– were associated with a steeper decline were also observed in the A+T+, but in that group higher levels were related to a steeper decline in A+T+ (i.e. these proteins were inversely associated with cognitive decline in both groups). Figure 3 illustrates this different relationship of protein levels with decline for A+T+ and A+T– subgroups for SEMA3C, a semaphorin-family member protein involved in regulating neuronal structure and synaptic plasticity^39^ and previously found to be associated with AD.^40^

An enrichment analysis for this specific subset of 87 proteins confirmed that dysregulation of synaptic plasticity pathways was driven in part by similar proteins (Supplementary Table 4). Replication analyses in ADNI on 1511 proteins that were measured in both datasets indicated that 540 out of 664 proteins in A+T– and 643 out of 718 proteins in A+T+ that were associated with decline on the MMSE in ADC were also measured in ADNI. In A+T−, 268 proteins (42% of 540) were significantly replicated in ADNI, while in A+T+ 152 proteins (28% of 643) were replicated. Considering that SOMAscan is a different technology than mass spectrometry, this indicates that these proteins can be picked up with both and their associations with decline replicate.

### CSF proteomic associations with progression to dementia in AT groups

**Figure 4:**
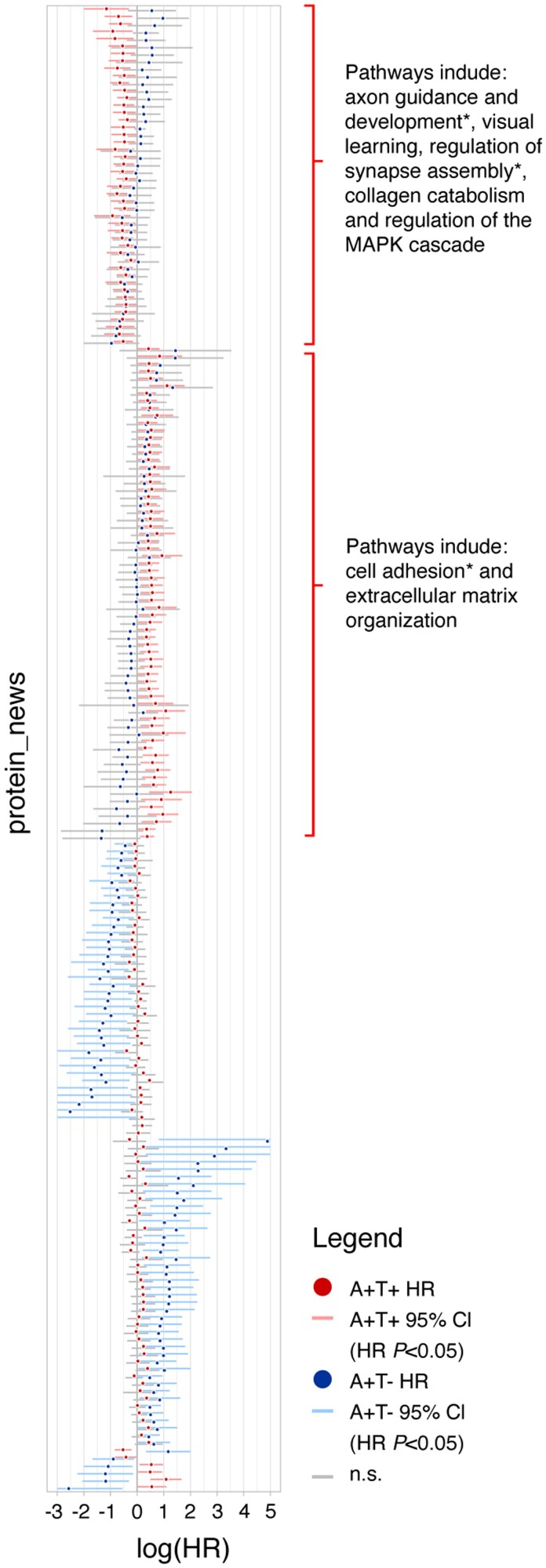
**Illustration of which proteins and their associated biological processes are related to clinical progression to dementia in A+T+ and A+T−.** Forest plot showing the log(HR) for progression to dementia for each protein in A+T+ (red) and A+T− (blue) MCI individuals. Lines indicating the 95% CI are coloured by group when the HR was significant (*P*-value <0.05), and grey when the HR was not significant. Notably, A+T– and A+T+ showed mostly unique proteomic signatures. Analyses were age, sex and education adjusted. An asterisk (*) behind a pathway indicates that that pathway was replicated in the Alzheimer's Disease Neuroimaging Initiative (ADNI) cohort. AT = CSF amyloid and tau status; CI = confidence interval; HR = hazard ratio; MCI = mild cognitive impairment.

**Figure 5:**
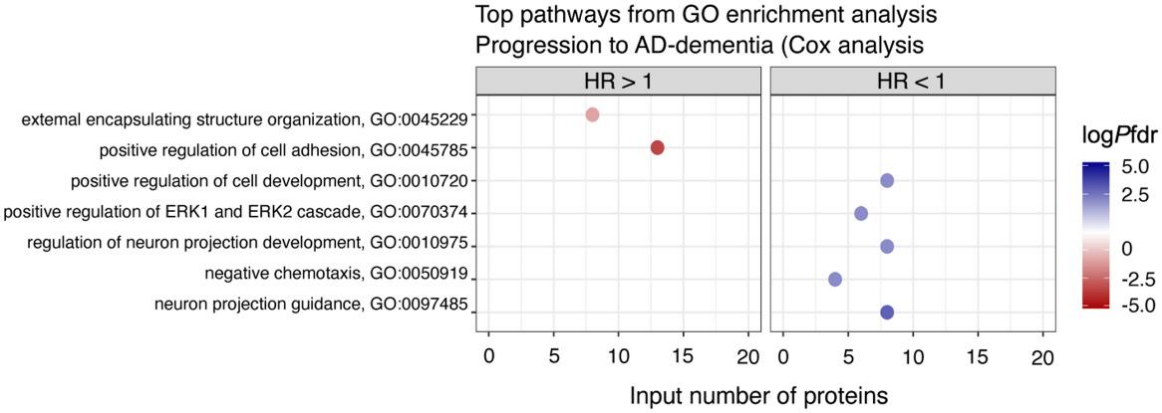
**GO pathway enrichment analysis.** Results from GO pathway enrichment analysis on proteins associated with progression to dementia in A+T+, stratified for direction of effect. GO:BP was used as the ontology source. Pathways with the highest *P*-value false discovery rate (pFDR) are shown, limited to one pathway per Panther Hierarchical group. The *x*-axis shows the number of proteins involved in each pathway that was associated with progression to dementia in each specific group. AT = CSF amyloid and tau status; GO:BP = Gene Ontology Biological Processes; HR = hazard ratio.

We then investigated which protein CSF levels at baseline were associated with subsequent clinical progression to dementia in A+T+ and A+T– individuals in ADC (detailed results in Supplementary Table 1). In A+T+, 119 (5%) proteins were associated with clinical progression (Fig. 4), of which 53 proteins were also associated with a steeper decline on the MMSE, including, e.g. KLK10 and CD44. For 71 of 119 proteins, higher levels predicted faster progression to dementia, and these were enriched for processes related to extracellular matrix (ECM) organization and cell adhesion (Fig. 5 and Supplementary Table 2), of which cell adhesion was replicated in ADNI. For 48 of 119 proteins, lower levels predicted faster progression, and these were associated with processes such as axon guidance and development, regulation of synapse assembly, collagen catabolism and regulation of the Mitogen-Activated Protein Kinase (APK) cascade. In ADNI, 80% of these pathways were replicated (Supplementary Fig. 1).

In A+T−, 87 (4%) proteins were associated with clinical progression (Fig. 4), of which 6 proteins overlapped with the 119 proteins found in A+T+. Additionally, 55 of 87 proteins were also associated with a steeper decline on the MMSE. For 43 of 87 proteins, higher levels including C-reactive protein (CRP) [HR (95% CI) 3.3 (1.3–8.2), *P* = 0.009], Neurofilament light (NEFL) [HR (95% CI) 4.3 (1.2–15.5), *P* = 0.03] and Guanosine diphosphate dissociation inhibitor 1 (GDI1) [HR (95% CI) 3.4 (1.2–9.4), *P* = 0.02] were associated with progression to dementia. For 44 of 87 proteins, lower levels predicted faster clinical progression. These were not associated with specific pathways. Last, we compared our results to those described in our previous study using the Olink platform.^8^ In the present study, we measured 76 of the proteins found to be predictive of progression to dementia, and found that the majority of these (52% in A+T+ to 68% in A+T−) had the same direction of effect sizes, including Cathepsin S (CTSS), TEK receptor tyrosine kinase (TEK) and Cluster of differentiation 46 (CD46). For 108 and 59 proteins that were associated with progression to dementia in A+T+ and A+T– individuals in ADC and also measured in ADNI, 13 (12%) and 7 (12%) proteins, respectively, were significantly replicated, which was less than the associations replicated with decline on MMSE scores. In A+T+, 67% of pathways were replicated in ADNI, including those related to aminoglycan metabolism and immune-related pathways. In A+T−, 58% of pathways were replicated in ADNI, including those related to aminoglycan metabolism, immune- and synaptic plasticity-related pathways (marked with an asterix in Fig. 2A, Supplementary Tables 2 and 3 and Supplementary Fig. 2).

## Discussion

In this study, we used an untargeted CSF proteomic approach to investigate the biological processes that may underlie interindividual differences in rates of cognitive decline in MCI with abnormal amyloid and normal and abnormal tau. Our main finding was that distinct proteomic signatures were found in A+T+ and A+T– MCI groups that were both associated with decline over time on the MMSE and progression to dementia. In MCI with A+T+ we found that mostly higher levels of proteins related to synaptic plasticity processes, and lower levels of proteins associated with the humoral immune system and actin filament organization were associated with steeper cognitive decline, while in MCI with A+T– we found that a large group of the same proteins were associated with slower cognitive decline. In that group we observed that higher levels of proteins indicative of blood–brain barrier impairment were also associated with steeper cognitive decline. Other processes associated with cognitive decline in A+T– included apoptotic signalling, glucose metabolism, and amyloid β formation and clearance pathways. In ADNI, which employed an orthogonal proteomics methodology, we found largely similar pathways associated with decline in A+T+ and A+T– compared with what we have found in ADC. Together these results indicate that CSF tau status is related to different underlying molecular processes contributing to cognitive decline, which suggests that treatments for slowing cognitive decline in AD may need tailoring on tau status.

In a previous study, we investigated CSF proteomic associations with progression to dementia in A+ MCI individuals, using Olink proximity extension assays, and found that lower levels of 126 proteins involved in immune system processes, neurodevelopmental pathways, signal transduction and neuronal death were all associated with a higher risk of progression to dementia.^8^ In the present study, we detected 76 of those proteins and found that many of these had the same direction of effect sizes. More importantly, we found enrichment for similar biological pathways as in our previous study. Because we took an untargeted proteomic approach, we were able to identify several other pathways that were associated with cognitive decline over time, and these included amyloid metabolism, ECM organization and proteoglycan metabolism. The proteins in this pathway included, e.g. APP, SMOC1 and COL11A1, which overlapped with proteins that belong to the matrisome protein module identified in tissue proteomics by Johnson *et al*.^12^ That module was enriched for ECM and glycosaminoglycan-related proteins and correlated strongly to Ad neuropathology and to cognitive functioning. We now observe in CSF that these processes seem to be predictive for future cognitive decline. This makes the ECM an interesting target for therapeutic intervention potentially affecting both neuropathology and cognition.

Furthermore, we observed that the proteins and pathways associated with decline in MCI A+ were dependent on tau biomarker status. According to the recently revised National Institute on Aging-Alzheimer's Association (NIA-AA) criteria, an A+T– profile fulfils the biological definition for AD and are at increased risk of progression of clinical symptoms.^21^ This is in line with the present study where we observed that individuals with MCI and an abnormal amyloid status and abnormal tau are at increased risk of developing dementia and had a steeper decline than MCI with abnormal amyloid and normal tau. Often, normal tau in MCI A+ is considered as indicating an earlier disease stage. However, our study suggests that this may reflect a different biological variant of Ad. This is in line with other studies reporting that in addition to tau tangles, CSF tau levels may also reflect other (patho)physiological processes that are disrupted in AD.^9,14,41^ For example, up to 70% of individuals with Ad dementia and CSF A+T– had high AD neuropathologic changes at post-mortem examination;^31,42^ and of those with repeated CSF measurements, most remained T– over time. Previous proteomics studies in tissue and CSF have further indicated that CSF tau and Braak tau stages correlate with different proteins related to distinct molecular processes.^11-13^ Amongst these studies, three focused on tissue proteomics measures in AD and revealed clusters of synapse- and neuron-related proteins that were associated with CSF tau levels and tau Braak stages. Furthermore, three cross-sectional CSF proteomics studies by Visser *et al*.^9^ and Tijms *et al.*,^14,15^ respectively, found that A+T+ individuals across the clinical continuum had increased levels of proteins related to aberrant neuronal plasticity, while these protein levels were decreased in A+T−, and that A+T– had signs of blood–brain barrier dysfunction. We now extend those findings with the observation that most of these processes were also related to cognitive decline over time. Importantly, individuals with MCI A+ with T+ or T– largely differed in which proteins were associated with a subsequent decline over time. For a part of the results similar processes seemed to be associated with cognitive decline in both T groups, but these were associated with different proteins. Furthermore, some processes were involved in both groups, but the CSF levels of those proteins had opposite associations with cognitive decline in AT subgroups. For example, neuronal plasticity-related processes were increased with a steeper decline in A+T+, while for the same proteins in those processes decreased levels associated with a steeper decline in A+T−. These results suggest that either too much (hyper-) or too little (hypo-) neuronal plasticity could contribute to cognitive decline. An important clinical implication of these results is that in addition to amyloid and tau markers other biomarkers may aid in providing a more accurate prognosis for MCI A+ individuals. In addition, our results imply that MCI A+ individuals may require treatments that are tailored to their underlying pathophysiological processes that are related to cognitive decline. Currently, treatments are being tested that target synaptic connectivity,^43^ and future studies should test in CSF if MCI A+T– and A+T+ groups may differ in their response to those interventions.

A potential limitation of our study might be that although the total sample size of MCI individuals with untargeted CSF proteomics as well as clinical cognitive follow-up available (*n* = 80) is one of the largest of its kind, subgroup sizes were relatively small. Still, we were able to include a second cohort for replication analysis, suggesting that the proteins and pathways that replicated are robust. In further studies we aim to keep increasing sample sizes. Although a large group of proteins was replicated, not all results were. This may reflect differences in the technique used to determine proteomics (MS versus SOMAscan or Olink) Future studies with both untargeted TMT-MS proteomics and proteomics measured on other platforms, such as SOMAscan and Olink, within the same individuals are necessary to further study platform-related effects.^44^ As such, we consider the number of proteins we replicated for our MMSE analysis quite considerable and these also point towards proteins that seem to be robustly related to cognitive decline. Another possibility is that differences in age between the cohorts may have contributed to differences between cohorts, as the ADC individuals were on average 10 years young than in ADNI. Still, the majority of biological processes related to cognitive decline and progression to dementia overlap between both cohorts suggesting that these pathways are mainly influenced by CSF tau status rather than age.

Lastly, it should be noted that the interpretation of high versus low CSF protein levels in relation to ongoing pathophysiological processes in the brain remains challenging. For example, a previous proteomics study that compared Ad-related protein changes across MS platforms and across brain and CSF compartments found that synaptic proteins generally showed discordant CSF and brain tissue expression levels (higher CSF levels, lower brain tissue levels), while glial and immune protein panels showed concordantly upregulated levels in both compartments.^13^ Future studies are necessary to investigate the factors that influence CSF protein levels, and how protein levels change over time. A strength of this study is that our cohorts were deeply phenotyped and had, especially for the clinical cohort, a long follow-up available.

In conclusion, we found that cognitive decline in MCI with A+T– or A+T+ was predicted by distinct CSF proteomic signatures that pointed towards involvement of different underlying pathophysiological mechanisms in these groups. These results imply that A+T– and A+T+ may require personalized treatment to slow down cognitive decline.

## Supplementary Material

awaf251_Supplementary_Data

## Data Availability

The data that support the findings of this study are available on request from the corresponding author. The data from the ADC are not publicly available due to privacy restrictions. ADNI data can be requested through their website.
