# Supplementary material for: CSF proteomic profiles related to cognitive decline in MCI A+ depend on tau levels
Source: Brain. 2025 Jul 8;148(12):4389–99. doi: 10.1093/brain/awaf251 (PMC12677021; doi:10.1093/brain/awaf251)
Supplement: awaf251_Supplementary_Data [file awaf251_supplementary_data.zip › brain-2024-03077-File008.pdf]

Supplementary table 1. Associations between baseline CSF protein levels and progression to AD-dementia (Cox regression analysis) and decline on MMSE over time (linear mixed model analysis) in ADC. When significant (P-value <0.05), HR and β estimates are shown in red when higher protein levels predict faster progression (i.e., HR>1, β protein*time <0) or are associated with worse cognition at baseline (β protein <0); and blue when lower protein levels predict faster progression (i.e., HR<1, β protein*time >0) or are associated with worse cognition at baseline (β protein >0).

Supplementary table 2. GO pathway enrichment analysis in ADC.

Supplementary table 3. Associations between baseline CSF protein levels and progression to AD-dementia (Cox regression analysis) and decline on MMSE over time (linear mixed model analysis) in ADNI. When significant (P-value <0.05), HR and β estimates are shown in red when higher protein levels predict faster progression (i.e., HR>1, β protein*time <0) or are associated with worse cognition at baseline (β protein <0); and blue when lower protein levels predict faster progression (i.e., HR<1, β protein*time >0) or are associated with worse cognition at baseline (β protein >0).

Supplementary table 4. GO pathway enrichment analysis in ADC, restricted to those proteins that showed an opposite effect on MMSE over time in A+T+ (B protein*time <0) and in A+T- (B protein*time >0).

Supplementary figure 1. Replication analysis in ADNI for GO pathway enrichment analysis on proteins associated with progression to dementia in A+T+.

Supplementary figure 2. Replication analysis in ADNI for GO pathway enrichment analysis on proteins associated with decline on MMSE over time in A+T+ and A+T- individuals with MCI.

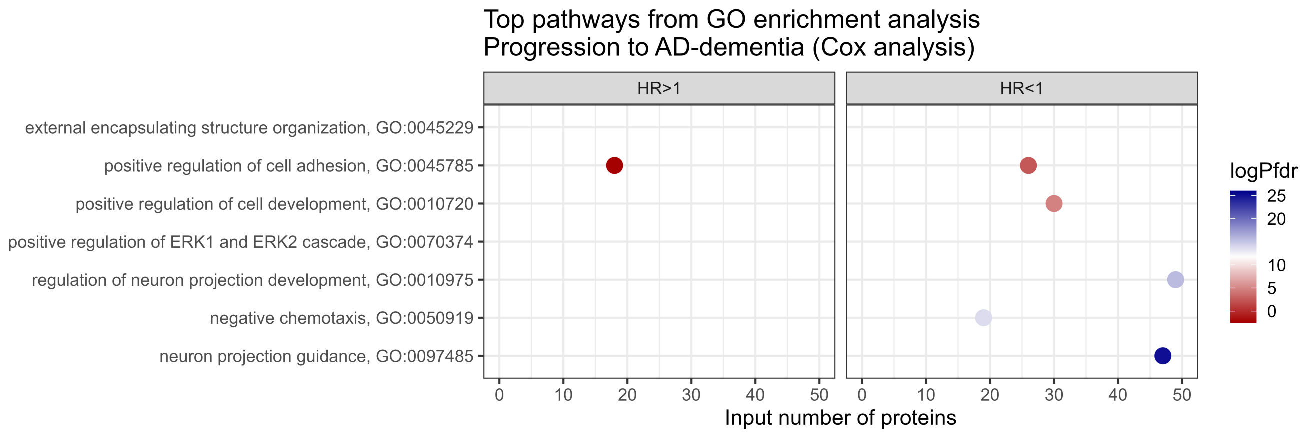
Top pathways from GO enrichment analysis
Progression to AD-dementia (Cox analysis)
HR>1
HR<1
external encapsulating structure organization, GO:0045229
positive regulation of cell adhesion, GO:0045785
positive regulation of cell development, GO:0010720
positive regulation of ERK1 and ERK2 cascade, GO:0070374
regulation of neuron projection development, GO:0010975
negative chemotaxis, GO:0050919
neuron projection guidance, GO:0097485
0 10 20 30 40 50
Input number of proteins
logPfdr
25
20
10
5
0

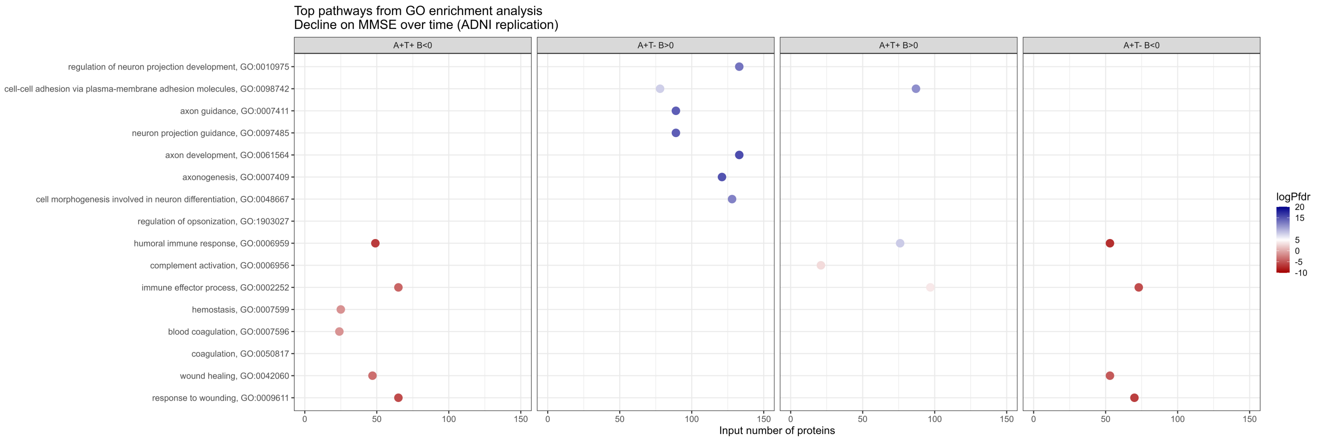
Top pathways from GO enrichment analysis
Decline on MMSE over time (ADNI replication)
A+T+ B<0
A+T- B>0
A+T+ B>0
A+T- B<0
regulation of neuron projection development, GO:0010975
cell-cell adhesion via plasma-membrane adhesion molecules, GO:0098742
axon guidance, GO:0007411
neuron projection guidance, GO:0097485
axon development, GO:0061564
axonogenesis, GO:0007409
cell morphogenesis involved in neuron differentiation, GO:0048667
regulation of opsonization, GO:1903027
humoral immune response, GO:0006959
complement activation, GO:0006956
immune effector process, GO:0002252
hemostasis, GO:0007599
blood coagulation, GO:0007596
coagulation, GO:0050817
wound healing, GO:0042060
response to wounding, GO:0009611
0 50 100 150
Input number of proteins
logPfdr
20
15
5
0
-5
-10
